# Cervical Cancer Stigma Among Caribbean Population: A Descriptive Paper

**DOI:** 10.3389/ijph.2024.1606725

**Published:** 2024-04-10

**Authors:** Kamilah Thomas-Purcell, Althea Bailey, Diadrey-Anne Sealy, Gaole Song, Kimlin Tam Ashing

**Affiliations:** ^1^ Department of Health Science, Nova Southeastern University, Ft. Lauderdale, FL, United States; ^2^ Department of Community Health and Psychiatry, University of the West Indies Mona, Kingston, Jamaica; ^3^ School of Public Health, Loma Linda University, Loma Linda, CA, United States; ^4^ Department of Population Sciences, Beckman Research Institute, City of Hope, CA, United States

**Keywords:** HPV, cervical cancer, the Caribbean, cancer stigma, health disparities

## Abstract

**Objectives:** Cervical cancer prevention practices are desperately low in the Caribbean. This study aims to describe the cervical cancer stigma and to evaluate the influence of the prevention practices among the Caribbean non-patient population in Jamaica, Grenada, Trinidad and Tobago.

**Methods:** A cross-sectional study involving 1,207 participants was conducted using a culturally trans-created Cancer Stigma Scale for the Caribbean context and supplemented with questions on cervical cancer and HPV/HPV vaccine knowledge and beliefs. Data collection took place online from October 2022 to March 2023.

**Results:** Participants are young, single, well-educated, and have stable financial resources. Over a quarter (26.4%) agreed women with cervical cancer are more isolated in their country. Almost half (47%) of respondents agreed cultural background plays a big part in how they feel about illness and getting well. One in six participants believe women with cervical cancer are treated with less respect than usual by others in their country.

**Conclusion:** Cancer stigma of cervical cancer exists in Jamaica, Trinidad and Tobago, and Grenada. Particularly, cultural background and social norms are closely linked to stigma.

## Introduction

Cervical cancer is the fourth most common cancer as well as one of the leading causes of death among women in the world [1]. Compared to women living in developed countries, women who live in developing countries are more vulnerable to cervical cancer due to the existing inequities in wealth, gender, and access to health services [2]. In the Americas, an estimated 35,700 deaths are caused by cervical cancer each year, and the majority (80%) of these cases are in Latin America and the Caribbean [3]. To improve women’s health, the World Health Organization (WHO) called for the elimination of cervical cancer, and the global goal is reaching and maintaining an incidence rate of below 4 per 100,000 women [4]. With efforts, the incidence and mortality have decreased in the past years; however, the rates in Latin America and the Caribbean are still higher than the threshold. Approximately 56,000 new cervical cancer cases were diagnosed in 2018 and 28,000 women lost their life early due to cervical cancer [5]. If current trends continue by 2030 in Americans, deaths caused by cervical cancer are predicted to increase to over 51,500, and over 89% of these deaths will occur in Latin America and the Caribbean [3].

Cervical cancer is indeed preventable. It is highly preventable and treatable, with up to 93% of cases being avoidable through effective screening and human papillomavirus (HPV) vaccination [6]. Approximately 99.7% of cervical cancer cases are attributed to chronic high-risk HPV infection. Getting vaccinated against HPV can provide robust protection for children and young adults, and even middle-aged individuals, against the most viral HPV infections and through regular screening tests, such as the Pap test (or Pap smear) and the HPV test [7, 8]. These tests can detect HPV infections and identify conditions that may potentially progress to cancer or pre-cancerous stages before becoming invasive cancer [8]. By implementing these prevention measures comprehensively and widely we have the potential to significantly reduce the incidence and mortality of cervical cancer and save thousands of lives.

However, despite the high burden, cervical cancer prevention practices are desperately low in the Caribbean. Overall, the current HPV vaccination rate in the Caribbean is below the PAHO targets and varies by country. Among all Caribbean countries, more than half have a national HPV vaccination coverage lower than 30% (among girls aged 9–14 years old) [9]. Evidence has shown high vaccine coverage in young girls (>80%) significantly reduces the risk of HPV infection in both girls and boys at a population level [9]. In addition, the cervical cancer screening rate is also low among adult women in the Caribbean with differences across countries [9, 10].

The reasons for insufficient cervical cancer prevention and early detection are complicated. One factor is cancer stigma, especially the stigma related to cervical cancer, that influences the uptake of prevention in the Caribbean. Cancer stigma is the negative attitude of society including those affected towards cervical cancer. Stigma is influenced by income, ethnicity, gender norms, culture, and type of cancer, and women suffer more from additional stigma when cancers (such as cervical cancer) are linked to their reproductive system and traditional gender roles [11].

There is limited research addressing the impact of stigma on cervical cancer prevention including Pap testing and HPV vaccination attitude and uptake in the Caribbean region. Therefore, this study aims to describe the cervical cancer stigma and to evaluate the influence of the prevention practices among the Caribbean non-patient population in Jamaica, Grenada, Trinidad and Tobago.

## Methods

### Study Design and Settings

This study applied a community-based participatory research approach. Grenada, Jamaica, and Trinidad and Tobago, among the many Caribbean nations characterized by low rates of pap testing and a high incidence of cervical cancer, served as foundational locations for future multinational studies. The Caribbean, also referred to as the West Indies, comprises numerous islands and archipelagos, totaling over 700 islands, spread across the Caribbean Sea. Geographically, Jamaica is situated within the Greater Antilles, Grenada is part of the Windward Islands, and Trinidad and Tobago (T&T) lies on the South American shelf. While T&T may not be considered part of the West Indies from a purely geographical perspective, it shares common historical and cultural ties with the other islands and is typically included in regional definitions. Academic researchers collaborated with community leaders in each country to conduct a cross-sectional quantitative study. Institutional Review Board (IRB) approval was obtained in each country.

### Participants

Survey responses were collected from 1,207 participants who were aged 18–85 and lived in Jamaica, Trinidad and Tobago, and Grenada for at least 10 years. All participants were English proficient and provided consent before answering any questions. Adults unable to provide informed consent or had a cognitive or psychiatric disorder that impaired their ability to safely participate in the study were excluded. At the end of the survey, participants had the option to be entered into a raffle to win a $50 USD gift card.

### Data Collection

Data collection took place online from October 2022 to March 2023. Participants provided their responses through the Research Electronic Data Capture system (REDCap), which is a secure, web-based application used for a wide variety of research studies.

### Measures

The survey used in this study was the culturally trans-created Cancer Stigma Scale (CASS) [12] for the Caribbean context and supplemented with questions on cervical cancer and HPV/HPV vaccine knowledge and beliefs. Demographic information (including age range, gender, sexual orientation, educational level, income range, employment status, and relationship status) was collected. Cervical cancer stigma was measured using updated questionnaire from four sub-scales including cancer stigma sub-scale, social impact sub-scare, health beliefs sub-scale, and quality of care sub-scale. Based on CASS, there are six domains to be measured in our questionnaire:

1) awkwardness: questions assessed whether people feel comfortable around women with cervical cancer e.g., employer/co-workers discriminate against persons with cervical cancer;
2) severity: questions assessed how people view the severity of diagnosing cervical cancer e.g., once you’ve had cancer you can never be “normal” again;
3) avoidance: questions measured whether people would keep distance from women with cervical cancer e.g., I would feel comfortable around someone with cancer;
4) personal responsibility: questions measured whether people believe an individual’s actions are considered to have contributed to their cervical cancer e.g., a person with cancer should be blamed for their condition;
5) policy opposition: questions measured how people think the government and the public to take actions towards the care and treatment of cancer patients e.g., more government funding should be spent on the care and treatment of those with cancer;
6) financial discrimination: questions assessed how people think about cancer patients benefiting from bank and insurance services e.g., banks should be allowed to refuse mortgage applications for cancer-related reasons.

In order to better understand the cultural and social influence on cancer stigma, the questionnaire also included questions such as “In my community and culture women with cervical cancer are looked down on,” “My culture believes that women with cervical cancer deserve less respect,” “Intimate partners reject women with cervical cancer,” etc.

In the cancer stigma sub-scale, we assessed participants’ belief in cervical cancer on a five-point Likert scale (“Strongly Agree,” “Agree,” “Not sure,” “Disagree,” “Strongly Disagree”) or a six-point Likert scale (“Strongly Agree,” “Moderately Agree,” “Slightly Agree Slightly,” “Disagree,” “Moderately Disagree,” “Strongly Disagree”).

For the social impact sub-scare and health beliefs sub-scale, participants’ responses were recorded on a five-point Likert scale (“Strongly Agree,” “Agree,” “Neither Agree nor Disagree,” “Disagree,” “Strongly Disagree”). Responses for the quality-of-care sub-scale were recorded as “Yes,” “No,” and “Don’t know.”

### Data Analysis

Categorical data were reported in frequency and percentage. Incomplete responses were not considered in this analysis. A cancer stigma variable was created. Cancer stigma responses were found to be not normally distributed. The normality test gave a significance of <0.001. Comparisons of skewness and Kurtosis gave values greater than 1.96 and/or less than −1.96. All statistical analysis was performed using IBM SPSS Statistics 29.0.0.

## Results

### Demographic Results

Demographic characteristics of the participants are summarized in Table 1, overall, 49% of respondents resided in Jamaica, 26% in Grenada, and 24% in Trinidad and Tobago. Almost 80% (78.2%) of participants were aged between 18 and 45 years and most respondents were female (81.4%). Many survey participants had a university-level education (44.2%) with a further 52.2% having a secondary, community college or trade/technical school level of education. More than half of respondents were employed on a full-time basis (53%) with 17% being students. Slightly, over half (51.0%) were single among participants. Overall, participants are young, single, well-educated, and have stable financial resources.

**TABLE 1:** Demographic characteristics of participants (Jamaica, Trinidad and Tobago, Grenada. 2023).

Characteristics	Frequency (%)
Residence Country	
Jamaica	589 (48.8)
Trinidad and Tobago	288 (23.9)
Grenada	316 (26.2)
Age	
18–25	403 (33.4)
26–35	282 (23.4)
36–45	258 (21.4)
46–55	129 (10.7)
56–65	91 (7.5)
65+	44 (3.6)
Gender	
Male	213 (17.6)
Female	983 (81.4)
Sexual Orientation	
Heterosexual/Straight	1,071 (88.7)
Relationship Status	
Currently married/living with someone	416 (34.5)
Divorced/lived with someone in the past	84 (7.0)
Single	615 (51.0)
Educational Level	
Secondary/High	290 (24.0)
Community College	230 (19.1)
Trade or Technical School	109 (9.0)
University	533 (44.2)
Employment Status	
Full-time	640 (53.0)
Part-time	125 (10.4)
Student	205 (17.0)
Retired	69 (5.7)

For the six domains assessed in the scale, the *Avoidance* domain has the highest mean score (5.641) and *Severity* has the lowest (3.681). Among all items, “I would feel irritated by someone with cancer” showed the highest mean score, which is 5.68. The mean scores of domains also show variety by country: the mean score of the *Avoidance* domain in Trinidad and Tobago is 5.774 which is higher than the overall mean score; the other two countries have slightly lower scores (Jamaica 5.574 and Grenada 5.630).

### Cultural/Societal Cervical Cancer Stigma

Response distribution is shown in Table 2.

**TABLE 2:** Cultural/societal cervical cancer stigma (Jamaica, Trinidad and Tobago, Grenada. 2023).

Cervical cancer stigma item	Strongly agree (%)	Agree (%)	Neither agree nor disagree (%)	Disagree (%)	Strongly disagree (%)
Employer/co-workers discriminate against persons with cervical cancer	2.7	8.0	22.6	30.2	36.5
Women with cervical cancer are treated with less respect than usual by others	3.6	12.3	21.0	34.1	29.0
Women with cervical cancer must keep it a secret	2.5	6.0	16.7	29.8	45.0
Women with cervical cancer are rejected by family members	1.8	6.5	20.4	34.5	36.7
Women with cervical cancer are rejected by intimate partners	5.5	20.3	38.7	19.6	16.0
A man will never marry a woman who has had cervical cancer	4.7	11.8	38.3	26.9	18.2
Intimate partners blame women for having cervical cancer	4.1	16.7	36.5	25.0	17.7
Women with cervical cancer should never disclose/tell a partner she has cervical cancer	2.3	3.1	7.3	31.7	55.6
My community’s negative view of cervical cancer may discourage women from getting the Pap smear (cervical cancer screening test)	7.6	22.0	18.6	27.7	24.2
My community’s negative view of cervical cancer may discourage women from getting further diagnosis after an abnormal Pap smear	7.5	21.2	18.6	29.7	22.9
The fear of family reaction and rejection may discourage women from getting further diagnosis after an abnormal Pap smear	8.0	27.6	15.7	27.7	21.0
It is unfair that society blames women with cervical cancer for their illness	39.6	29.3	11.9	8.4	10.8
In my community and culture women with cervical cancer are looked down on	4.1	11.4	28.5	29.9	26.0
Women with cervical cancer are more isolated	5.1	21.3	32.3	22.6	18.6
My family believes that women with cervical cancer deserve less respect	2.0	43.6	9.1	27.4	57.9
My culture believes that women with cervical cancer deserve less respect	1.9	5.4	14.3	30.7	47.8
In my family cervical cancer is viewed as shameful	1.9	2.7	9.9	27.6	57.8
In my community cervical cancer is viewed as shameful	2.7	6.5	18.3	28.8	43.7
My cultural background plays a big part in how I feel about my illness and getting well	14.7	32.3	19.0	18.8	15.2
Cervical cancer can be caused by being unclean (not washing often during menstrual period or after sexual intercourse)	4.5	14.4	33.8	23.1	24.2

#### Community/Society

In this domain, questions were asked to measure how community, society, and culture would influence people’s attitudes and feelings toward patients with cervical cancer.

Over a quarter (26.4%) of participants agreed women with cervical cancer are more isolated in their country. Less than 10% believed women with cervical cancer deserve less respect in their culture and approximately 10% agreed cervical cancer is viewed as shameful in their community. Meanwhile, almost half (47%) of respondents agreed cultural background plays a big part in how they feel about illness and getting well.

In addition, one in six of participants believe women with cervical cancer are treated with less respect than usual by others in their country; 8.5% think women with cervical cancer must keep it a secret. Although over half (68.9%) of our participants agreed that it is unfair that society blames women with cervical cancer for their illness, there are still about one in six reported women with cervical cancer are looked down on in their community and culture. Almost, one in five (18.9%) believed cervical cancer can be caused by being unclean (not washing often during the menstrual period or after sexual intercourse).

Regarding cervical cancer screening, almost, one in three of the participants agreed that the community’s negative view of cervical cancer may discourage women from getting the Pap smear (cervical cancer screening test) or further diagnosis after an abnormal Pap smear, which indicated cultural background and social norms play a very important role in cervical cancer screening and treatment.

#### Interpersonal Relationship

In this domain, questions were asked to measure people’s attitudes and feelings toward patients with cervical cancer in familial, intimate, and other interpersonal relationships.

Cervical cancer stigma exists in intimate relationships. 16.5% believed a man will never marry a woman who has had cervical cancer while 20.8% thought intimate partners would blame women for having cervical cancer. In addition, 25.8% of the participants believed women with cervical cancer are rejected by intimate partners; 5.4% agreed that women with cervical cancer should never disclose/tell a partner she has cervical cancer.

For familial relationships, less than 10% (8.3%) of the participants agreed women with cervical cancer are rejected by family members. Reactions of family members also influenced cervical cancer screening: more than a quarter (25.6%) indicated the fear of family reaction and rejection may discourage women from getting further diagnosis after an abnormal Pap smear. Not many people (5.6%) reported their family believes that women with cervical cancer deserve less respect and about 5% stated cervical cancer is viewed as shameful in their family.

Besides, 1 in 10 of the participants agreed employer/co-workers would discriminate against persons with cervical cancer. These numbers suggest women with cervical cancer still face unfair criticism, especially in intimate relationships.

## Discussion

This is one of the first studies examining cancer stigma within the Caribbean region. This study reinforces the existing literature that culture influences critical sources of cancer stigma including community, familial, and intimate relations to cervical cancer stigma. One in five respondents agreed that it is shameful for a woman to have cervical cancer and that a woman deserves less respect because of her disease. One explanation for this stigma is the fact that HPV is sexually transmitted and causes cervical cancer. HPV infection is regarded as a symbol of promiscuity or unfaithfulness since having multiple sexual partners is a risk factor for HPV [13]. However, often women are at increased risk for HPV infection due to exposure from their male partners [14]. Importantly, almost everyone who is sexually active has been exposed to some strain of HPV; even if that person only has one partner [15, 16].

From our descriptive results, about 80% of the participants endorsed that society’s cancer stigma is unfair, but they reported persistent community, familial, and intimate relation stigma associated with cervical cancer. Cancer stigma was associated with a low likelihood that women with cervical cancer would seek support from their family members and intimate partners [17]. This study found that one in four participants agreed women with cervical cancer are rejected by intimate partners; over a quarter of participants believed fear of family reaction and rejection may discourage women from getting further diagnosis after an abnormal Pap smear. Previous research has stated that social support is positively associated with preventive practices for cervical cancer [18].

Our findings indicate that although people do not have a strong personal stigma related to cervical cancer, most of them admit the stigma exists in their community and society. Changing the community, familial and intimate-relations stigmas must be prioritized to improve prevention and screening practices as well as diagnostic, therapeutic, and survivorship care. Public media campaigns emphasize that cervical cancer is highly treatable and even curable when detected at early stages. Health practitioners should consider patient/client referral for cervical cancer screening and HPV vaccination, and couple and family-centered education as well. Lack of support from close family members and partners would negatively impact women’s care-seeking behaviors, which will cause increased disease burden for both individuals and society. To enhance prevention, screening, diagnosis, treatment, and survivorship care for cervical cancer, it is crucial to address and challenge the social stigmas associated with community, family, and intimate relationships. Public awareness campaigns should highlight the high treatability and potential curability of cervical cancer when detected early. Healthcare providers should actively encourage patients to undergo cervical cancer screening and HPV vaccination, and they should also promote education that involves couples and families.

Additionally, it is important to recognize that the absence of support from close family members and partners can have a detrimental impact on women’s willingness to seek care. This lack of support can lead to a heavier disease burden for both individuals and society as a whole. Therefore, prioritizing efforts to eliminate these stigmas and foster a supportive environment is essential.

Another effective strategy is to involve community health workers (CHWs) who are from the local community. They can play a vital role in educating residents and motivating families of eligible age to get HPV vaccines. Additionally, CHW’s can encourage adult women to undergo regular screening tests. Leveraging CHWs is particularly advantageous because they share a similar background with the target population, making it easier to engage with local residents and convey the importance of cervical cancer prevention.

### Limitations

This study has several limitations. Firstly, the majority of our respondents were women due to the convenience sample used in this preliminary study. To obtain more comprehensive and well-rounded results, it is essential to include a larger representation of men in the study. Additionally, the questionnaire utilized in this study did not inquire whether respondents had ever received the HPV vaccine or undergone cervical cancer screening tests, which represents another limitation. Furthermore, a significant portion of the data was collected online, potentially biasing the sample towards a more educated population.

### Summary

In summary, this preliminary study aimed to provide fundamental insights into cancer stigma. Future research efforts should prioritize the development of interventions involving multi-stakeholders including clinicians, researchers, advocates, survivors, and policymakers, to effectively eradicate stigmas and lessen the burden of cervical cancer in the Caribbean region. Crucially, national public health departments should take the lead in crafting health policies and prevention strategies. These strategies should encompass comprehensive nationwide cervical cancer education campaigns that take stigma-related factors into account. Such initiatives are essential to mitigating stigma and enhancing screening and vaccination rates. And ultimately reduce the burden of cervical and other HPV-related cancers within Caribbean communities through multi-level interventions.
